# Intumescent Flame Retardant Mechanism of Lignosulfonate as a Char Forming Agent in Rigid Polyurethane Foam

**DOI:** 10.3390/polym13101585

**Published:** 2021-05-14

**Authors:** Weimiao Lu, Jiewang Ye, Lianghai Zhu, Zhenfu Jin, Yuji Matsumoto

**Affiliations:** 1 School of Engineering, Zhejiang A&F University, Hangzhou 311300, China; luweimiao@stu.zafu.edu.cn (W.L.); yjw0418@zafu.edu.cn (J.Y.); 2020104061022@stu.zafu.edu.cn (L.Z.); 2 Graduate School of Agricultural and Life Sciences, The University of Tokyo, 1-1-1 Yaoyi, Bunkyo-ku, Tokyo 113-8657, Japan; amatsumo@mail.ecc.u-tokyo.ac.jp

**Keywords:** lignosulfonate, intumescent flame retardant, char forming agent, flame retardancy mechanism

## Abstract

Intumescent flame retardants (IFR) have been widely used to improve flame retardancy of rigid polyurethane (RPU) foams and the most commonly used char forming agent is pentaerythritol (PER). Lignosulfonate (LS) is a natural macromolecule with substantial aromatic structures and abundant hydroxyl groups, and carbon content higher than PER. The flame retardancy and its mechanism of LS as char forming agent instead of PER in IFR formulation were investigated by scanning electron microscopy, thermogravimetric analysis, limiting oxygen index testing and cone calorimeter test. The results showed LS as a char forming agent did not increase the density of RPU/LS foams. LOI value and char residue of RPU/LS foam were higher than RPU/PER and the mass loss of RPU/LS foam decreased 18%, suggesting enhanced thermal stability. CCT results showed LS as a char forming agent in IFR formulation effectively enhanced the flame retardancy of RPU foams with respect to PER. The flame retardancy mechanism showed RPU/LS foam presented a continuous and relatively compact char layer, acting as the effect of the flame retardant and heat insulation between gaseous and condensed phases. The efficiency of different LS ratio in IFR formulation as char forming agent was different, and the best flame retardancy and thermal stability was obtained at RPU/LS1.

## 1. Introduction

Rigid polyurethane (RPU) foams, which are prepared by polymerization of isocyanates with polyols, are generally used as an insulating material in construction, refrigeration, and piping/tubing industries. However, the inherently high flammability of RPU foams has caused a large number of fire-related casualties [1,2]. Recently, the intumescent flame retardant (IFR) has aroused great attention due to it being more environmentally friendly than the traditional halogen-containing flame retardant, and has therefore been widely used to improve flame retardants in RPU foams [3,4,5]. IFR formulations consist of three parts, namely char forming agent, acid source, and gas source, which provide materials flame retardancy by changing the mode of decomposition into a condensed phase mechanism [6,7,8,9].

The flame retarding mechanism of IFR is based on the charred layer acting as a physical barrier, which slows down heat and mass transfer between gaseous and condensed phases [10,11]. It is well-known that a char forming agent is the most important component in IFR formulation, forming an intumescent porous char layer [12,13]. The char forming agents commonly used in intumescent formulations for RPU foams are polyols such as pentaerythritol (PER), starch, sucrose, mannitol, sorbitol, ethylene glycol, et al., which is easily dehydrated by the acid to form a char layer [14,15,16,17,18]. PER is the most commonly used char forming agent, which contains 4 hydroxyl groups and 44% carbon content [18,19]. Nevertheless, because of the low molecular weight of PER, there are certain limitations, namely, poor flame retardant efficiency, low thermal stability, weak water resistance, and easy migration to the surface of the material [20,21,22]. Renewed interest is emerging for the sustainable development of flame retardants with bio-based macromolecules for composites [23,24].

Lignin is a natural macromolecule with substantial aromatic structures and abundant hydroxyl groups, and the carbon content of lignin is higher than that of PER [25,26]. The aromatic structure of lignin could provide thermal stability. The high carbon content of lignin could produce a large amount of char residue upon heating at elevated temperatures, and the abundant aliphatic and phenolic hydroxyl groups could provide good reacting sites with the acid to form a char layer [27,28,29,30]. Reti et al. [14,31] prepared flame-retardant PLA composites by incorporating lignin and starch with APP as IFR with 10% loading amount. The results showed that PER can be substituted by bio-resources and the flame retardation of the PLA/APP/lignin and PLA/APP/starch composites were greatly improved compared with that of pure PLA. Cayla et al. [32] selected Kraft lignin as the carbon source, combined with APP, in the manufacture of PLA texture. The results found that the flame retardant performance of PLA texture was better than that of PLA with Kraft lignin or APP alone due to the synergistic effect between Kraft lignin and APP. Zhang et al. [33,34,35] prepared composites containing IFR composed of lignin and APP to enhance the flame retardancy of PLA. These researchers have proposed lignin as a bio-based char forming agent with great potential for application duo to its abundance in nature [36]. Moreover, lignin can be dissolved in diethylene glycol (DEG), which is a kind of polyol, reacting with isocyanates to form polyurethane [37].

Industrial lignin, which is produced on a large scale, is divided into sodium lignosulfonate (LS), Kraft lignin (KL), solvolys lignin (SL), and hydrolysis lignin (HL). Lignosulfonate is a kind of industrial lignin obtained by the sulfite pulping process and used as char forming agent in the preparation of green polyurethanes.

In this study, the possibility of lignin as a char forming agent instead of PER, and the mechanism of lignin as a char forming agent in IFR formulation for RPU foams were investigated. Moreover, the effect of different lignin ratios as char forming agent in the IFR system were studied.

## 2. Materials and Methods

### 2.1. Materials

Lignosulfonate (LS: Mw:11700, Mn:3900, Mw/Mn:3.0) [38], which was derived with the softwood sulfite pulping process, was supplied by Yanbian Shixian Bailu Papermaking Co., Ltd. Methylene diphenyl diisocyanate (MDI-400, NCO = 32%) was purchased from Wanhua Polyurethane Co., Ltd. (Yantai, China). Ammonium polyphosphate (APP: (NH_4_PO_3_)n, n > 1000) and Melamine (MEL) were obtained from Macklin Biotech Co., Ltd. (Shanghai, China). Pentaerythritol (PER) was purchased from Hangzhou Lanbo Co., Ltd. (Hangzhou, China), Diethylene glycol (DEG, OH = 32%) from Lusen Chemicals Co., Ltd. (Linyi, China), Di-n-butyltindilaurate (DBTDL) from Shanghai Lingfeng Chemicals Co., Ltd. (Shanghai, China), and silicon oil from Dow Corning Co., Ltd. (Shanghai, China). All reagents commercially available were used without further purification.

### 2.2. Rigid Polyurethane (RPU) Foam and IFR Formulations

#### 2.2.1. Preparation of RPU Foam

The RPU foams was prepared as following: 40 g DEG was mixed with 0.3 g distilled water as the foaming agent, 0.04 g DBTDL as the catalyst, 4 g silicon oil as foam stabilizer, and intumescent flame retardants (IFR) stirred together until homogenization; then 100 g MDI-400 was added into the mixture under continuous stirring. When it began to foam, the mixture was immediately poured into the mould and cured at room temperature for 24 h.

#### 2.2.2. The Ratio of IFR Formulations

The ratio of acid source/gas source/char forming agent of IFR was 3:1:1, namely 10.2 g APP as acid source, 3.4 g MEL as gas source, and 3.4 g char forming agent was added into the above RPU foams as a flame retardant. The char forming agent was 3.4 g PER, 1.7 g PER/1.7 g LS, and 3.4 g LS, respectively, and the RPU foams were designated as RPU/PER, RPU/PER/LS, and RPU/LS, respectively (Table 1). The density of RPU/PER, RPU/PER/LS, and RPU/LS foams were kept stable, ranging from 57.5 to 58.0 kg/m^3^ (Table 1).

#### 2.2.3. The Best Ratio of LS as Char Forming Agent in IFR Formulation

Different ratios of LS as a char forming agent in IFR systems were designed as Table 2, to investigate the best ratio of LS as a char forming agent in RPU foams. 10.2 g APP as acid source, 3.4 g MEL as gas source, and different contents of LS as char forming agents were added into the above RPU foams as a flame retardant. When the LS content was 1.7 g, 3.4 g, 5.1 g, 6.8 g, 10.2 g, and 13.6 g, the ratio of APP:MEL:LS of IFR formulations was 3:1:0.5, 3:1:1, 3:1:1.5, 3:1:2, 3:1:3, and 3:1:4, respectively, and the RPU foams were designated as RPU/LS0.5, RPU/LS1, RPU/LS1.5, RPU/LS2, RPU/LS3, and RPU/LS4, respectively. The density of RPU/LS foams ranged from 57.3 to 58.2 kg/m^3^ (Table 2).

### 2.3. Characterizations

The density of RPU foams was calculated according to GB/T 6343-2009. Sample size was 100 mm × 100 mm × 3 mm.

Limiting oxygen index (LOI) testing was carried out on a JF-3 oxygen index instrument (Nanjing Jiangning Analytical Instrument Co., Ltd., Nanjing, China) according to GB/T 2406.2-2009. The samples (100 mm × 10 mm, 10 mm thick) were held vertically in an oxygen index measurement system. Ten samples were tested to obtain average values.

The cone calorimeter test (CCT) of RPU foams was measured using an FTT UK cone calorimeter instrument (Stanton Redcroft Limited, East Grinstead, UK) according to ISO5660 and ASTME1354-94 standard. Each RPU foam specimen (100 mm × 100 mm × 30 mm) was irradiated at a heat flux of 35 kW/m^2^, and the test time was 600 s.

Thermogravimetric (TG) analysis was performed on a NETZSCH TG209 thermal analyzer (The NETZSCH Group, Selb Germany). About 5~10 mg of each sample was scanned from room temperature to 800 °C at a scanning rate of 10 °C/min under nitrogen atmosphere at a flow rate of 20 mL/min.

Scanning electron microscopy (SEM) of RPU foams after CCT test was studied by TM3033 (Hitachi High Technologies Corporation, Tokyo, Japan) to observe the micro-morphology of the residue chars tested by CCT, and the specimens were gold-sputtered with a conductive layer; the accelerated voltage was 5 kV.

## 3. Results and Discussion

### 3.1. The Effect of LS as a Char Forming Agent in IFR Formulation

To achieve flame retardancy via an intumescent process, three ingredients are necessary, namely an acid source (precursor for catalytic acidic species), a char forming agent, and a gas source [10,18]. The effectiveness of the char forming agent depends on carbon content and hydroxyl groups. The char forming agent commonly used in the intumescent formulations for RPU foams is PER, which contains 4 hydroxy groups and 44% of carbon content [18,19]. Lignin contains abundant hydroxyl groups and around 60% carbon content [25]. Table 1 shows that even with a higher carbon content of LS, the density of RPU/PER, RPU/PER/LS, and RPU/LS were similar to each other (Table 1), suggesting that LS as a char forming agent instead of PER did not increase the density of RPU foams. Moreover, LS could dissolve in DEG due to abundant hydroxy groups [17]. Therefore, using LS as a char forming agent instead of PER could avoid exudation and water solubility problems associated with PER [39].

**Figure 1:**
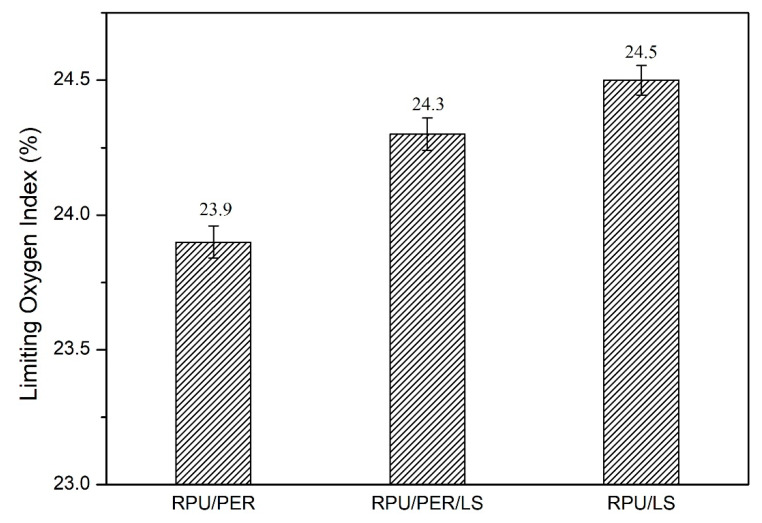
The effect of LS as a char forming agent on LOI.

The LOI value is an important parameter for comparing the flammability of polymeric materials. As seen from Figure 1, the LOI value of RPU/PER sample was 23.9%. When 50% or 100% of PER was substituted by LS in IFR formulation, the LOI value gradually increased by 1.7% and 2.5%, respectively, suggesting that LS as a char forming agent was superior to PER in IFR formulation for RPU foams.

**Figure 2:**
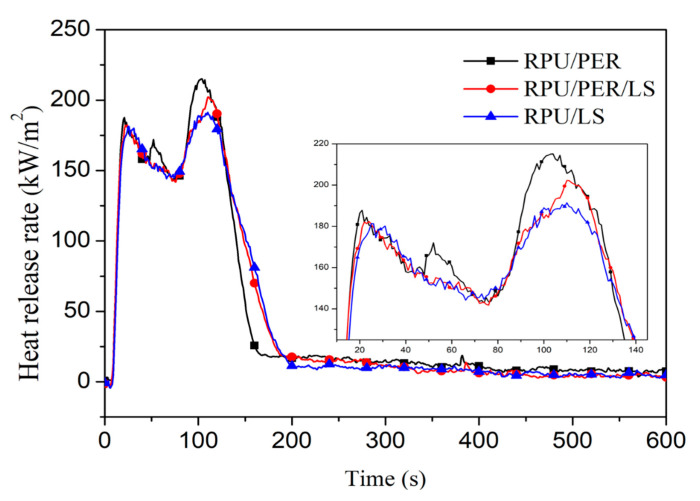
HRR curves of RPU/PER, RPU/PER/LS, and RPU/LS foams.

CCT was performed to further evaluate the combustion behavior and flame retardant performances of RPU foams in a realistic fire environment [3]. The heat release rate (HRR) curves for RPU/PER, RPU/PER/LS, and RPU/LS showed typical IFR formulation for RPU foams (Figure 2), which exhibits two peaks. The first peak is assigned to the ignition and spread of flames on the surface of the foams and then the intumescent char layer protecting the foams, and the second peak is explained by the destruction of the intumescent structure and the formation of a carbonaceous residue [11]. The HRR of the RPU/LS foam was lower than that of RPU/PER, suggesting that LS as a char forming agent exhibited more effective flame retardancy.

The peak of heat release rate (pHRR) is the most important parameter of a given fire scenario that reflects the tendency of a flame retardant system towards fire propagation [40]. The higher the value of pHRR, the greater the danger of a fire hazard [3,41]. As Figure 2 and Table 3 show, the pHRR value of RPU/LS and RPU/PER/LS foams decreased 11% and 6%, compared to that of RPU/PER foam, respectively. The time to peak heat release rate (tpHRR) of RPU/LS foam was pushed by 6 s, compared to the RPU/PER foam, suggesting that the fire retardancy of RPU foams improved after PER was substituted by LS. In addition, the total heat release (THR) value of RPU/LS foam (27.4 MJ/m^2^) was lower with respect to RPU/PER, providing further evidence that the flame retardancy of RPU/LS foam was superior to that of RPU/PER foam (Table 3). The fire growth rate index (FGRI) is calculated by dividing pHRR by tpHRR, which can estimate both the predicted fire spread rate and the size of a fire. The higher the FGRI value, the faster is the spread of the flame spread and flame growth [40,42]. The RPU/LS foam exhibited good flame-retardant behavior. When PER was substituted by LS 100% or 50%, the values of FGRI of RPU/LS and RPU/PER/LS foams decreased by 19% and 14%, compared to the value of RPU/PER foam (Table 3), suggesting that LS as a char forming agent in IFR formulation more effectively enhanced the flame retardancy of RPU foams.

Notably, the total smoke rate (TSR), specific extinction area (SEA), effective heat of combustion (EHC), peak of carbon monoxide yield (pCO), mean carbon monoxide yield (mCOY), and mean carbon dioxide yield (mCO_2_Y) values were used to characterize the flame-retardant effect of LS as a char forming agent in the gaseous phase. It could be seen that the TSR value of RPU/LS was much lower than that of RPU/PER, suggesting that LS as a char forming agent in the IFR system could effectively suppress smoke and volatile gas production (Table 4). The SEA presents the relationships between volatilization property and smoke emission [43]. The SEA value of RPU/LS was reduced by 20% in comparison to RPU/PER foam, which indicated that the smoke-generating capacity of each unit mass and the contribution degree of volatiles produced by RPU/LS foams were much lower than that of the RPU/PER foam.

The mCO_2_Y value was similar between RPU/LS and RPU/PER formulation, while the mCOY value of RPU/LS increased compared to the RPU/PER system, suggesting that more incomplete combustion products were produced during combustion due to cutting off of the transfer between the heat source and foam material by the carbon char layer. Conclusions of suppression of smoke and volatile gases production were in agreement with Hou et al. [42], who reported that more incomplete combustion product (CO) produced during combustion demonstrates a strong flame retardant effect in the gaseous phase. This would be due to the lignin aromatic structure, which easily formed radicals in the early stage of combustion, and the radicals from lignin consumed oxygen radicals, resulting in more incomplete combustion product produced during combustion [44]. As a consequence, the pCO value from RPU/LS was 79% lower compared to RPU/PER foam (Table 4), indicating that RPU/LS displayed more effective flame retardant effects in the gaseous phase. Carbon monoxide (CO) is a toxic volatile gas, which is the main cause of death during fire. LS as a char forming agent can more effectively suppress smoke production and the toxicity of volatile gases, therefore exhibiting an improvement in flame retardant performance. The EHC value indicates the efficiency of combustion of the volatiles produced from thermal degradation of foams, which can be used to understand the mechanism of fire degradation of foams [7,24]. The EHC value of RPU/LS foam was 6% lower compared to RPU/PER foam, which represented a low amount of volatile gas generated from RPU/LS foam. This result was in accordance with SEA, mCOY, mCO_2_Y, and TSR (Table 4). This may be due to the fact that LS as a char forming agent formed a stable and compact char layer, which retarded the transfer of heat and volatile gas, resulting in improvement in flame-retardant properties. LS, acting as a char forming agent, displayed a more effective role in reducing fire hazards than that of PER in the gaseous phase.

The CCT results further show that the peak of mass loss rate (PMLR) and specific mass loss rate (SMLR) of RPU/LS foam decreased by 46% and 14% with respect to RPU/PER foam, respectively. The time to peak mass loss rate (t_PMLR_) of RPU/LS foam was pushed by 26 s, compared to the RPU/PER foam (Table 5). These results indicated that the RPU/LS foam showed enhanced thermal stability, compared to the RPU/PER foam. Moreover, the char residue of RPU/LS foam after CCT increased 24% with respect to the RPU/PER foam, which provided evidence for the enhanced thermal stability of LS as a char forming agent instead of PER.

**Figure 3:**
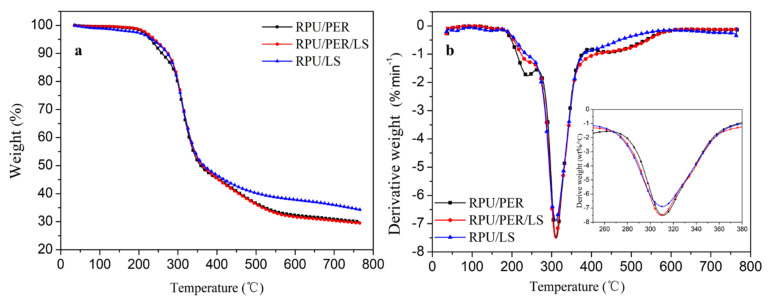
TG (**a**) and DTG (**b**) curves of RPU/PER, RPU/PER/LS, and RPU/LS foams under nitrogen atmosphere.

The TG analysis results further supported the CCT conclusions. The mass loss after TG analysis of the RPU/LS foam decreased by 18% with respect to RPU/PER foam, suggesting an enhanced effect on thermal stability by LS (Table 5). The RPU/PER, RPU/PER/LS, and RPU/LS foams presented similar thermal degradation decomposition behavior, showing two steps of thermal degradation—the first step appeared around 210 °C with mass loss less than 5%, which would be due to evaporation of water and decomposition of low molecular compounds (Figure 3). The second step appeared at 311 °C with mass loss over 50%. The mass loss of RPU/LS and PU/PER were 50.7% and 61.9% in the range of 277–356 °C and 273–361 °C of initial (T_onset2_) and stop (T_final2_) temperature, respectively (Table 5). Compared with the RPU/PER foam, the char residue of RPU/LS foam increased by 15% (Table 5). The lower mass loss and higher char yield of RPU/LS foam would be due not only to the high carbon content, but also the aromatic structural characteristics of lignin, which could produce radicals, resulting in reducing degradation rate and increasing char yield, and providing further evidence for enhanced thermal stability of LS as a char forming agent instead of PER [38]. These results indicate that compared to PER, LS was more efficient as a char forming agent in IFR for RPU, which was in agreement with CCT analysis. 

### 3.2. The Flame Retardant Mechanism of LS as a Char Forming Agent in IFR Formulation

**Figure 4:**
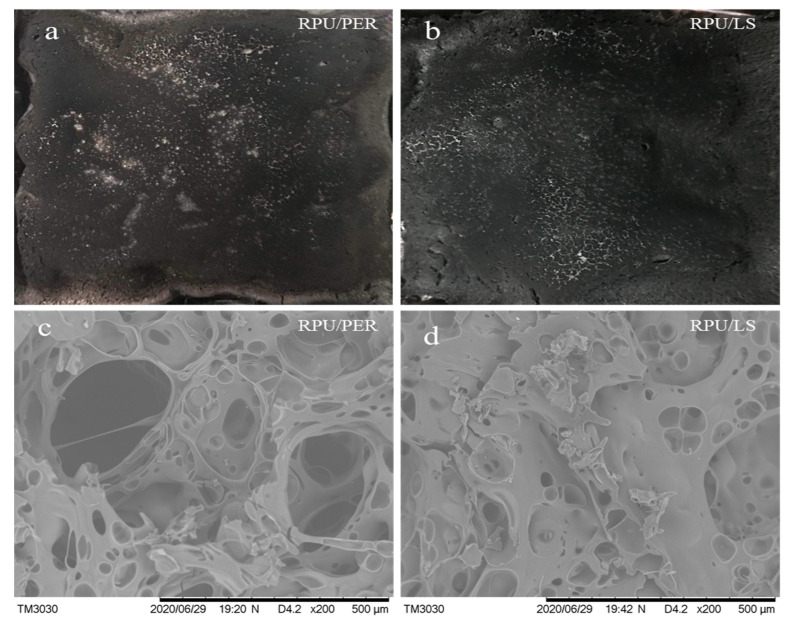
Digital photos (**a**,**b**) and SEM micrograph (**c**,**d**) of char residues for RPU/PER and RPU/LS foams.

The residual appearances of RPU/LS and RPU/PER foams after CCT are shown in Figure 4a,b. It can be seen that the surface of the RPU/LS foam showed a continuous and relatively compact char layer, which was similar to that of the RPU/PER foam. The results indicated that LS as a char forming agent was able to form a good and coherent char layer, which could slow down the heat transfer and spread of flames, thus protecting the underlying materials from further burning.

The SEM micrograph of char residues of RPU/LS and RPU/PER foams are shown in Figure 4c,d. The char residue of RPU/LS foam showed compact, continuous, and spongy-like structures, which could resist both mass and heat transfer (Figure 4c). Undoubtedly, this kind of char structure was conducive to prevent the release of combustible gas and retard the degradation of underlying material [41,45]. In contrast, the RPU/PER foam exhibited heterogeneous char residue with many big holes (Figure 4d), suggesting that the underlying foam substrate was not effectively prevented from degradation during combustion. Compared to PER, the morphology and structure of the char layer of LS as a char forming agent was more effective in creating a barrier, a protective shield against mass and heat transfer (Figure 4).

**Figure 5:**
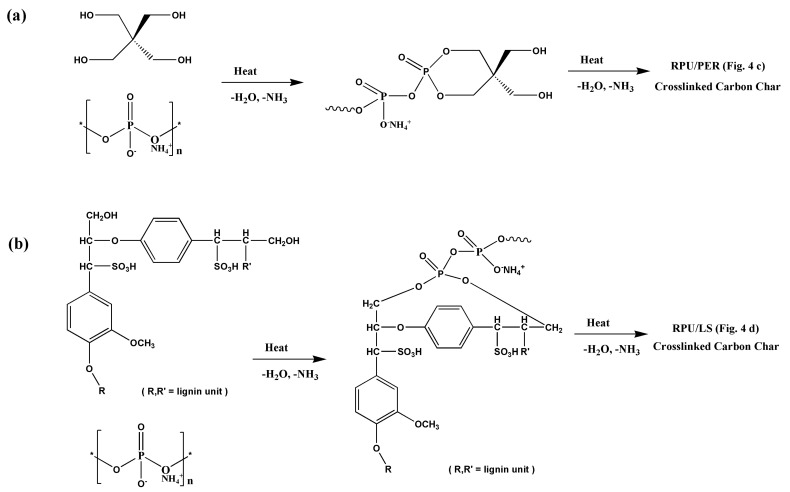
Flame retardant mechanism of PER/APP/MEL (**a**) and LS/APP/MEL (**b**) IFR formulation in RPU foam.

Based on the analysis results, the flame retardant mechanism is illustrated in Figure 5. The protection mechanism is based on the char layer acting as a physical barrier, which slows down heat and mass transfer between the gas and condensed phases [11,46]. A classic intumescent system is ammonium polyphosphate (APP) as acid source, PER as char forming agent, and melamine as gas formation source [18,19]. The function of APP was to catalyze the dehydration and elimination of NH_3_ by forming degradation products such as orthophosphates and phosphoric acid. The reaction of APP and its degradation products with the char forming agent (PER) took place with the formation of ester mixtures, and the carbonization process then took place with the formation of a cross-linked carbon char layer (Figure 5a) [18]. On the other hand, when LS was used as a char forming agent, the hydroxyl groups of LS reacted with APP and its degradation products, resulting in the aromatic ether mixture and leading to a carbon-carbon unsaturated cross-linked carbon char layer (Figure 5b) [47]. The substantial aromatic structures and high carbon contents of LS was able to produce a large amount of char residue. Meanwhile, the aromatic structure of LS was able to produce stable radicals, i.e., п-radicals in which the unpaired electrons trapped radicals, such as HO•,H•, and induced the carbonization process, resulting in a much more compact and carbonaceous char layer with high flame retardancy and thermal stability. At the same time, incomplete combustion and less amounts of non-combustible volatile products such as NH_3_, H_2_O produced and swelled the cross-linked carbon char layer, which effectively worked on thermal insulation and gas insulation effects on foams, preventing them from further decomposing and impeding the transport of mass and energy [32,33,48].

### 3.3. The Best Ratio of LS as a Char Forming Agent in IFR Formulation

**Figure 6:**
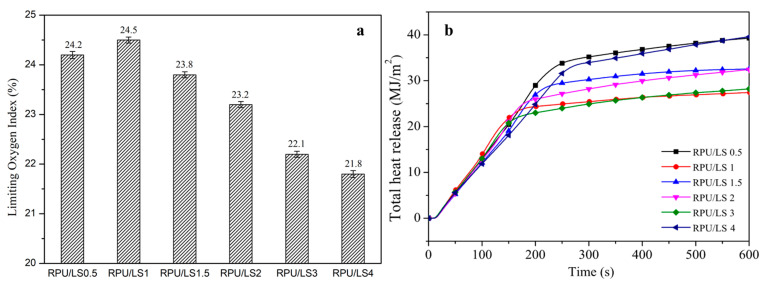
LOI (**a**) and THR (**b**) curves of RPU/LS foams.

From the above results, LS as a natural aromatic macromolecule with high carbon content and abundant hydroxyl groups could be used as a char forming agent instead of PER in IFR formulation for RPU foams. A suitable LS ratio in IFR formulation as a char forming agent can improve the flame retardancy of RPU/LS foams based on the LOI value. The LOI value of RPU/LS1 foam was achieved at 24.5%, which was the highest compared to other different LS ratios for RPU foams (Figure 6). The THR curves of RPU/LS foams showed similar trends while the THR values of RPU/LS1 foam were the lowest (Figure 6). Moreover, the HRR, EHC, and PMLR values of RPU/LS1 foam were relatively the lowest, which were 27.4 MJ/m^2^, 46.0 kW/m^2^, 17.0 MJ/kg, and 0.26 g/s, respectively. The time to peak carbon dioxide (t_PCO2_) of RPU/LS1 foam was pushed by 478 s compared with that of RPU/LS0.5 foam (Table 6). These results indicate the efficiency of different LS ratios; the best flame retardancy was obtained for RPU/LS1. This would be due to the synergic effects of char forming agent (LS) and APP in the IFR system, which mainly acts in the condensed phase by formation of a continuous and compact char structure and inhibited further combustion of the combustible gas produced by thermal decomposition.

**Figure 7:**
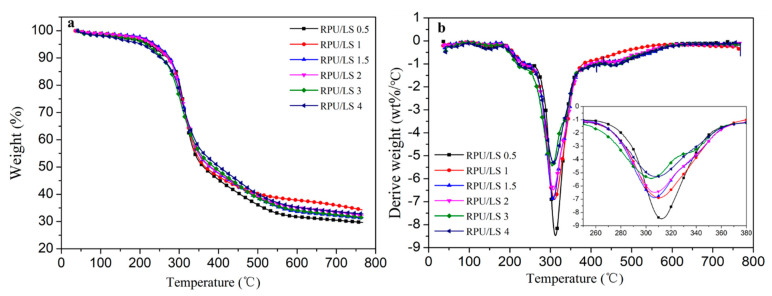
TG (**a**) and DTG (**b**) curves of RPU/LS foams under nitrogen atmosphere.

The RPU/LS foams also showed two-step thermal degradation with the first step mass loss less than 4% and the second degradation step mass loss more than 50% (Figure 7). Among all the RPU/LS foams, the char obtained at 800 °C and the T_max2_ of RPU/LS0.5 foam were the lowest, while the mass loss in the second degradation step achieved the highest of 59.3%. The RPU/LS1 foam exhibited the best thermal stability. The mass loss in the second degradation step was the lowest, while the T_max2_ and char residue were the highest, which was 50.6%, 311 °C, 34.3%, respectively. With the increasing LS ratio, the thermal stability of foams progressively decreased (Table 7). RPU/LS1 foam exhibited the best thermal stability, indicating that a suitable LS ratio as a char forming agent was an important factor in IFR formulation, significantly affecting the thermal stability of foams.

## 4. Conclusions

Carbon content of LS is higher than that of PER, while LS as a char forming agent did not increase the density of RPU/LS foams. The LOI value of the RPU/LS foam was higher than that of RPU/PER, suggesting that LS was superior to PER as a char forming agent in IFR formulation for RPU foams. The mass loss of RPU/LS foam decreased by 18%, suggesting enhanced thermal stability in comparison with RPU/PER foam. Char residue of RPU/LS foam was higher than that of RPU/PER foam, and CCT results showed that with respect to PER, LS as a char forming agent in IFR formulation effectively enhanced the flame retardancy of RPU foams. The flame retardancy mechanism showed that RPU/LS foam presented a continuous and relatively much more compact char layer with continuous and spongy-like structures, highlighting the effect of the flame retardant, heat insulation, and protecting the inner matrix materials between the gaseous and condensed phases. From the above results, LS as a natural aromatic macromolecule with high carbon content and abundant hydroxyl groups could be used as a char forming agent instead of PER in IFR formulation for RPU foams. A suitable LS ratio in IFR formulation as a char forming agent can improve the flame retardancy of RPU/LS foams based on the LOI value. The CCT and TG analyses indicate the efficiency of different LS ratios; the best flame retardancy was obtained for RPU/LS1.

## Figures and Tables

**Table 1:** The formulation of IFR for RPU foams.

Sample	APP (g)	MEL (g)	PER (g)	LS (g)	Density (kg/m^3^)
RPU/PER	10.2	3.4	3.4	-	58.0
RPU/PER/LS	10.2	3.4	1.7	1.7	57.6
RPU/LS	10.2	3.4	-	3.4	57.5

**Table 2:** The ratio of LS as a char forming agent in IFR formulations for RPU foams.

Sample	APP (g)	MEL (g)	LS (g)	APP:MEL:LS Ratio	Density (kg/m^3^)
RPU/LS0.5	10.2	3.4	1.7	3:1:0.5	57.3
RPU/LS1	10.2	3.4	3.4	3:1:1	57.5
RPU/LS1.5	10.2	3.4	5.1	3:1:1.5	57.6
RPU/LS2	10.2	3.4	6.8	3:1:2	57.8
RPU/LS3	10.2	3.4	10.2	3:1:3	58.0
RPU/LS4	10.2	3.4	13.6	3:1:4	58.2

**Table 3:** Effect of LS as a char forming agent on heat transfer between the gaseous and condensed phases.

Sample	HRR (kW/m^2^)	pHRR (kW/m^2^)	tpHRR (s)	THR (MJ/m^2^)	FGRI (kW/m^2^·s)
RPU/PER	46.1	215.1	104	28.4	2.1
RPU/PER/LS	45.7	202.2	110	27.8	1.8
RPU/LS	45.0	191.4	110	27.4	1.7

HRR: heat release rate; pHRR: peak of heat release rate; tpHRR: time to peak heat release rate; THR: total heat release; FGRI: fire growth rate index.

**Table 4:** Effect of LS as a char forming agent on gas transfer between the gaseous and condensed phases.

Sample	TSR (m^2^/m^2^)	SEA (m^2^/kg)	EHC (MJ/kg)	pCO (kg/kg)	mCOY (g/kg)	mCO_2_Y (kg/kg)
RPU/PER	717.4	430.2	18.1	0.14	0.55	2.05
RPU/PER/LS	765.9	444.3	16.7	0.18	0.49	1.95
RPU/LS	700.4	343.8	17.0	0.03	0.68	2.03

TSR: total smoke release; SEA: specific extinction area; EHC: effective heat combustion; pCO: peak of carbon monoxide; mCOY: mean carbon monoxide yield; mCO_2_Y: mean carbon dioxide yield.

**Table 5:** Effect of LS as a char forming agent on mass loss.

Sample	CCT Results				TG Results (Second Step of Mass Loss)				
	PMLR (g/s)	SMLR (g/s.m^2^)	t_PMLR_ (s)	Char_600 s_ (g)	T_onset2_ (°C)	T_max2_ (°C)	T_final2_ (°C)	Mass Loss (wt%)	Char_800 °C_ (wt%)
RPU/PER	0.48	11.1	9	2.1	273	311	361	61.9	29.9
RPU/PER/LS	0.30	10.3	6	2.1	274	309	364	63.0	29.5
RPU/LS	0.26	9.6	35	2.6	277	311	356	50.7	34.3

PMLR: peak of mass loss rate; SMLR: specific mass loss rate; t_PMLR_: time to peak mass loss rate; Char_600 s_: char residue at 600 s after cone calorimeter test; T_onset2_: initial temperature; T_max2_: peak temperature; T_final2_: stop temperature of second mass loss step; Char_800 °C_: char residue at 800 °C from TG analysis.

**Table 6:** Effects of LS ratios as a char forming agent for the flame retardancy of RPU/LS foams.

Sample	THR (MJ/m^2^)	HRR (kW m^2^)	EHC (MJ/kg)	PMLR (g/s)	t_PCO2_ (s)
RPU/LS0.5	39.3	65.9	17.4	0.35	118
RPU/LS1	27.4	46.0	17.0	0.26	596
RPU/LS1.5	32.6	54.6	16.9	0.44	563
RPU/LS2	32.5	54.5	19.3	0.61	205
RPU/LS3	28.2	47.4	18.8	0.29	366
RPU/LS4	39.6	66.5	18.5	0.29	565

THR: total heat release; HRR: heat release rate; EHC: effective heat combustion; PMLR: peak of mass loss rate; t_PCO2_: time to peak carbon dioxide.

**Table 7:** TG data of LS ratios as a char forming agent on the thermal stability of RPU/LS foams.

Sample	T_max1_ (°C)	Mass Loss1 (wt%)	T_max2_ (°C)	Mass Loss2 (wt%)	Char_800__°C_ (wt%)
RPU/LS0.5	181	2.4	302	59.3	29.6
RPU/LS1	174	2.4	311	50.6	34.3
RPU/LS1.5	169	2.5	307	58.3	31.2
RPU/LS2	173	2.7	307	59.8	32.3
RPU/LS3	173	2.9	304	57.5	31.4
RPU/LS4	183	2.4	308	55.4	32.8

T_max1_ and T_max2_: maximum degradation temperature in the first degradation step and the second degradation step; Mass loss1 and Mass loss2: the mass loss in the first step and second step; Char_800 °C_: char residue at 800 °C from TG analysis.

## Data Availability

The data presented in this study are available on request from the corresponding author.
